# Health-related quality of life in children with haemophilia in China: a 4-year follow-up prospective cohort study

**DOI:** 10.1186/s12955-019-1083-3

**Published:** 2019-02-06

**Authors:** Heng Zhang, Jie Huang, Xiaoyan Kong, Gaoxiang Ma, Yongjun Fang

**Affiliations:** 1 Department of Hematology and Oncology, Children’s Hospital of Nanjing Medical University, Nanjing, 210008 China; 2 Department of anesthesiology, Children’s Hospital of Nanjing Medical University, Nanjing, 210008 China; 3 School of Traditional Chinese Pharmacy, China Pharmaceutical University, Nanjing, 211198 China

**Keywords:** Haemophilia, Health related quality of life (HRQoL), The Canadian Haemophilia outcomes–kids’ life assessment tool (CHO-KLAT), Prognosis

## Abstract

**Background:**

Health-related quality of life (HRQoL) has been brought up for decades in haemophilia patients. However, no data to date are available about HRQoL in children with haemophilia using long-term follow up data. This nearly 4-year follow-up study aimed to assess the long-term HRQoL of haemophilia children.

**Methods:**

A prospective cohort study among 42 children with haemophilia and their parents was conducted in August 2014 in a children’s hospital; follow-up was completed in January 2018. Primary endpoint was the change in patient HRQoL evaluated by Canadian Haemophilia Outcomes–Kids’ Life Assessment Tool (CHO-KLAT) from baseline to year 4; secondary endpoint was the impact of bleeding rates, physical activity restriction, financial burden and treatment (prophylaxis vs on-demand treatment) on HRQoL, as well as the impact of treatment on event-free survival.

**Results:**

Totally 42 patients (mean age, 5.48[SD, 4.63] years) and 42 parents were included. 38 families completed 4-year follow up. Patients reported a small increase in HRQoL from baseline to year 4. The mean scores of child self-report and parent proxy report of CHO-KLAT at baseline were 60.69 (SD = 20.28) and 61.01 (SD = 12.14), respectively. Scores at follow-up were 64.69 (SD = 13.71) and 65.33 (SD = 15.78), respectively. Haemophilia patients without physical activity restriction, living in urban areas, and receiving prophylactic treatment and home injection, had higher average values for HRQoL scores than the others. Bleeding rates were proportionally negatively correlated with HRQoL. Patients who had received prophylactic treatment had better event-free survival.

**Conclusions:**

Haemophilia decreased HRQoL of patients, but this effect weakened after 4 years. HRQoL of children is influenced by severity of haemophilia, bleeding rates, physical activity restriction, financial burden and treatment. Prophylactic treatment is a key factor contributing to event-free survivor prognosis and the optimal form of therapy for childhood haemophilia.

## Introduction

Haemophilia, characterized by a bleeding disorder which primarily affects boys, is caused by an inherited deficiency of factor VIII (haemophilia A) or factor IX (haemophilia B) [1] and is associated with some symptoms such as spontaneous bruising, mucosal bleeding, joint bleeding, epistaxis, and severe or even fatal bleeding events like intracranial hemorrhages [1–3]. The prevalence of haemophilia A is currently estimated to be about 1 case per 5000 male live births annually, while 1 case of haemophilia B occurs per 30,000 [1]. Haemophilia is classified as severe (< 1%), moderate (1–5%), or mild (5–40%) with concentration of the factor measured [4]. This classification predicts the general risk of bleeding, a guide to the optimum management strategy and outcome predictions. Repeated joint bleeding can cause severe joint damage and pain, leading to disability [5]. Bleeding issues can be addressed as they occur (on-demand) or treatment can be given regularly to prevent bleeding (prophylaxis) [2, 5, 6]. Frequent hospital visits, injections and limited (sports) participation are common impairments for haemophilia patients, most of whom worry about bleeding, the need for invasive procedures (like blood tests and intravenous therapy) and the risk of permanent disability. Consequently, this disease and its treatment impact patients’ health-related quality of life (HRQoL).

The concept of HRQoL was raised decades ago in haemophilia [7–10], and recent research outcomes emphasize the importance of considering HRQoL issues during decision making procedures surrounding the treatment of haemophilia [11–16]. HRQoL assessment provides a comprehensive vision of the patients’ subjective feelings of function and well-being, and supports evaluation of therapeutic effect [10, 13, 16]. The Canadian Haemophilia Outcomes–Kids’ Life Assessment Tool (CHO-KLAT) is a well-known disease-specific measure of HRQoL for childhood haemophilia patients [8, 17, 18]. Socio-Economic Context (SEC) module is a questionnaire aiming at capturing the impact of social economic context on individual families [16]. We used the two measures to evaluate patients’ HRQoL and explore the effect different local governments and types of insurance coverage have on haemophilia families [15].

There has been mounting evidence on the assessment of the quality of life (QoL) in haemophilia patients. However, a long period follow-up of HRQoL for childhood haemophilia remains rare. This prospective cohort study assessed the effect of haemophilia on patient-reported QoL from baseline to year 4. We also explored the effect of bleeding rates, physical activity restriction, financial burden and treatment (prophylaxis vs on-demand treatment) on HRQoL, as well as the impact of treatment on event-free survival. The study hypotheses were that haemophilia would affect patients’ QoL, and HRQoL with haemophilia would have association with bleeding rates, financial burden and treatment.

## Methods

### Patients

A total of 42 subjects with haemophilia were recruited along with their parents from Nanjing Children’s Hospital affiliated with Nanjing Medical University, between August 2014 and January 2018 Parents (or legal guardians of the subjects) were interviewed in person to collect clinical information. Among these 42 subjects, 37 had haemophilia A and the others had haemophilia B. The median age of subjects interviewed was 5.48 (SD = 4.63). Patients with cognitive impairments, limited literacy skills, or other chronic illnesses such as cerebral palsy, diabetes, chronic immune thrombocytopenia, rheumatological disease and asthma were excluded from this study. According to the subjects’ disease history, no subjects were excluded. Besides, those who did not complete CHO-KLAT during the needed time-frame were considered ineligible for this research. Children aged 7 years old and older were asked to complete self-report versions of CHO-KLAT and self-report versions of SEC with little or no interruption from parents.

### Measures

This is a 4-year prospective cohort follow-up study. Their early and recent responses were considered baseline data and follow-up data respectively. Events considered in event-free survival analysis were death, developing target joints (a target joint is a joint in which three or more spontaneous bleeds have occurred within a consecutive 6 month period [5]) and severe bleeding during the observation. CHO-KLAT is a disease-specific HRQoL measure that can be used in both clinical and home settings and has been updated to version 2.0 [8, 18]. It has been validated in several countries, including Canada, France, Germany, the Netherlands, Spain, the UK and China [14, 19] and consists of 2 questionnaires: one for children aged from 5 to 18 (child self-report), the other for parents on behalf of children aged from 2 to 18 (parent proxy report). This Chinese version includes all 35 original items from the CHO-KLAT. Scores have a range of 0 (worst) to 100 (best). There are no normative values of the CHO-KLAT. Higher scores mean better HRQoL. Children aged 7 years old and older, if literate, were asked to complete a self-report CHO-KLAT. Also, their parents were asked to complete a proxy CHO-KLAT on their behalf. The Chinese version has an additional unique SEC module, which consists of 9 questions covering information about stigma, opportunities for stable employment and access to treatment due to cost, etc. SEC scores have the same range as those of the original CHO-KLAT. The questionnaires were handed in hard copy to parents and patients in the clinic. Data were entered into an electronic database by researchers. All data were double entered to ensure accuracy. Data checks and cleaning were also performed. If more than 1/4 of answers were not given in one report, its score would not be shown and it was rejected for the research.

### Statistical analysis

Statistical analyses here were performed using Statistical Package for Social Science (version 22.0; SPSS). Qualitative variables were described in number and percentages and mean ± SD, if quantitative. Preliminary analyses used paired sample t-tests to evaluate differences between baseline and follow-up patient-rated HRQL and one-sample t-tests to compare patient HRQL to normative values. ANOVA tests were used when comparing quantitative data between 2 groups or more. The Spearman coefficients were used when studying the relationship (direction and power) of quantitative variables simultaneously. The correlations between mean scores and bleeding rates were performed using correlation analysis. Kaplan–Meier survival curves were plotted, and the groups compared using the log-rank test to ascertain the relationship between treatment and prognosis. Cox regression model adjusted age, region of residence, severity and Family injection was used to calculate the *P* value. The events we considered in KM analysis were death, target joints developing and severe bleeding. Two-sided *P* values were selected and *P* < 0.05 was considered statistically significant.

## Results

### Patients’ characteristics

The frequency distributions of all subjects of the selected variables (the demographic, clinical, and biochemical data at enrolment, as well as treatment during follow-up) are provided in Table 1. 18 cases (42.9%) had a history of severe bleeding; 15 (35.7%) suffered from intracranial hemorrhages and 11 (26.2%) had a single target joint at the time of recruitment while 4 cases had multiple target joints. 8 cases had restricted physical activity at baseline. The restricted physical activity is defined as impact on daily life activities in the past one month because the QoL questionnaires are filled according to participants’ situation during the past one month. None of the patients were known to have an inhibitor at enrolment. All the 4 mild hemophilia boys, 16 of 24 children with moderate disease, and 5 of 14 with severe disease received an on-demand therapy, while the others were subjected to prophylactic treatment at medium dose (15 - 30 U/kg, two or three times a week). During the follow-up, 4 patients were lost (2 cases had a history of target joints). Only 1 case suffered from severe bleeding (urinary system) and 4 cases developed target joints. 4 cases with target joints at baseline got better (the target joint stopped experiencing symptoms). Of all cases at the endpoint, 13 cases had target joints; however, merely 7 cases had restricted physical activity. One patient had an inhibitor.

**Table 1:** Frequency distribution of selected variables in Haemophilia patients

	Patients (*n* = 42)	
Variables	n	%
Age patients (years), mean (SD)	5.48 (4.63)	
Region of residence		
Rural	17	40.5
Urban	25	59.5
Diagnosis		
Haemophilia A	37	88.1
Haemophilia B	5	11.9
Severity		
Severe	14	33.3
Moderate	24	57.2
Mild	4	9.5
Type of treatment		
On-demand treatment	25	59.5
Prophylactic treatment	17	40.5
Family injection		
No	30	71.43
Yes	12	28.57

### CHO-KLAT and SEC scores of children

The results of this comparison are shown in Table 2. At baseline, all parents on behalf of their children completed the parent version of the CHO-KLAT and SEC while only 12 patients were old enough to complete it independently. The mean scores for the CHO-KLAT child/proxy report were 60.69 (SD = 20.28) and 61.01 (SD = 12.14) respectively. For the SEC, the mean scores were 60.62 (SD = 14.32) and 56.43 (SD = 12.59) respectively. There were no significant differences between the scores of 12 proxy and child self-reports in CHO-KLAT or SEC (*p* = 0.440 and *p* = 0.275, respectively). At follow-up, 38 parents completed the scales. 18 children were old enough to complete the child self-reports scales independently. The mean score for the CHO-KLAT child self-report and parent proxy report were 64.93 (SD = 13.71) and 65.33 (SD = 15.78) respectively. The child/proxy report SEC scores were 65.09 (SD = 15.36) and 61.62 (SD = 17.46). Statistical significance was not achieved between the scores of 18 proxy and child self-reports in CHO-KLAT or SEC (*p* = 0.694 and *p* = 0.522, respectively). Scores at follow-up were higher than those at baseline in both CHO-KLAT and SEC but no statistical significance was observed.

**Table 2:** Scores of CHOKLAT and SEC for Child/Proxy groups

HRQoL Scales	N	Baseline Scores			N	Follow-up Scores		
		Mean values (SD)	MINI	MAX		Mean values (SD)	MINI	MAX
CHOKLAT								
Child self-report	12	60.69 (20.28)	25.78	93.38	18	64.69 (13.71)	33.87	83.93
Parent proxy report	42	61.01 (12.14)	25.76	79.00	38	65.33 (15.78)	19.70	86.67
SEC								
Child self-report	12	60.62 (14.32)	36.11	90.63	18	65.09 (15.36)	38.89	87.50
Parent proxy report	42	56.43 (12.59)	31.25	93.75	38	61.62 (17.46)	30.56	96.88

No major relation was found between the two types of haemophilia and the scores of the child/proxy reports. Urban patients had significantly higher scores than rural patients in CHO-KLAT. Considering urban patients had higher insurance coverage than rural patients (85% vs 30–75%), financial status would be an explanation for the above difference, which was also proved by the SEC results in Table 3. As with severity of disease, the statistical difference appeared only in the CHO-KLAT parent proxy report group (*p* = 0.037) and SEC child self-report group (*p* = 0.040) at baseline. The mean scores of CHO-KLAT and SEC child/proxy reports in the prophylactic group were significantly higher than those in the on-demand group, especially for the parent proxy reports for CHO-KLAT and SEC. Patients who had received home injection got higher QoL at follow-up.

**Table 3:** Comparison between Scores of Child/Proxy Relation to Clinical Characteristics and Treatment of Haemophilia

Clinical Characteristics and Treatment of Haemophilia		Baseline Scores								Follow-up Scores							
		CHOKLAT Child self-report		CHOKLAT Parent proxy report		SEC Child self-report		SEC Parent proxy report		CHOKLAT Child self-report		CHOKLAT Parent proxy report		SEC Child self-report		SEC Parent proxy report	
		Mean SD	*P* ^1^	Mean SD	*P* ^1^	Mean SD	*P* ^1^	Mean SD	*P* ^1^	Mean SD	*P* ^1^	Mean SD	*P* ^1^	Mean SD	*P* ^1^	Mean SD	*P* ^1^
Type of haemophilia	A	60.12 (21.17)	0.763	61.13 (11.34)	0.861	60.44 (15.01)	0.898	55.70 (11.65)	0.309	63.56 (13.23)	0.154	66.42 (14.46)	0.279	65.61 (15.67)	0.570	62.80 (17.95)	0.290
	B	66.96 (−)		60.10 (18.73)		62.5 (−)		61.88 (19.06)		83.93 (−)		58.12 (23.54)		56.25 (−)		53.82 (12.53)	
Severity	Severe	44.23 (25.50)	0.135	56.94 (14.96)	**0.037**	44.44 (7.35)	**0.040**	51.74 (7.52)	0.086	58.65 (15.80)	0.364	64.58 (17.35)	0.274	63.60 (15.53)	0.949	62.33 (19.04)	0.207
	Moderate	71.67 (11.21)		64.92 (9.20)		63.53 (5.74)		60.13 (14.74)		69.47 (13.25)		68.09 (15.20)		66.41 (17.50)		64.03 (16.99)	
	Mild	55.19 (22.02)		51.76 (8.98)		70.95 (20.34)		50.70 (1.39)		64.20 (10.24)		54.18 (9.14)		64.67 (14.52)		47.05 (6.12)	
Region of residence	Rural	48.41 (17.80)	0.073	55.62 (14.00)	**0.016**	56.03 (13.63)	0.374	51.18 (12.04)	**0.024**	62.19 (13.48)	0.505	63.99 (15.44)	0.695	56.60 (11.10)	**0.031**	54.44 (9.09)	0.052
	Urban	69.46 (18.09)		64.67 (9.30)		63.89 (14.91)		60.00 (11.90)		66.69 (14.27)		66.11 (16.24)		71.88 (15.32)		65.80 (19.86)	
Physical activity restriction	No	70.69 (12.20)	**0.007**	62.70 (10.42)	0.062	63.89 (13.40)	0.283	57.66 (11.87)	0.196	66.31 (11.92)	0.365	66.31 (15.60)	0.430	65.95 (15.28)	0.670	62.15 (16.70)	0.701
	Yes	40.70 (19.01)		53.82 (16.67)		54.07 (15.74)		51.22 (15.04)		59.03 (19.85)		61.01 (17.07)		62.07 (17.62)		59.28 (21.88)	
Type of treatment	On-demand	52.64 (19.26)	**0.045**	56.81 (12.02)	**0.005**	60.06 (16.60)	0.860	51.13 (9.44)	**0.000**	61.49 (15.40)	0.167	58.82 (18.02)	**0.003**	57.26 (11.86)	**0.000**	51.41 (13.06)	**0.000**
	Prophylactic	76.78 (11.17)		67.19 (9.63)		61.72 (10.32)		64.24 (12.81)		71.10 (6.57)		73.37 (6.72)		80.73 (7.24)		74.23 (13.65)	
Home injection	No	59.96 (22.40)	0.870	60.68 (13.00)	0.901	60.02 (16.57)	0.850	56.26 (13.60)	0.671	62.18 (15.43)	0.221	60.97 (16.95)	**0.010**	59.35 (13.62)	**0.006**	55.02 (15.27)	**0.000**
	Yes	62.14 (18.26)		61.23 (11.34)		61.81 (10.44)		58.22 (11.72)		71.21 (3.27)		74.79 (6.47)		80.00 (7.85)		75.90 (13.05)	

*P* ≤ 0.05: significant

*P* ≤ 0.01: highly significant

^1^Tested by ANOVA test

Value in bold indicate significant *P* value

The mean of annual bleeding rate (ABR) and annual joint bleeding rate (AJBR) were 7.29 (SD = 9.73; ranging from 0 to 48), 2.86 (SD = 5.82; ranging from 0 to 24) at baseline, respectively, and 8.50 (SD = 10.16; ranging from 0 to 40), 4.50 (SD = 7.64; ranging from 0 to 40) at follow-up, respectively. The amount of change during this follow-up study in ABR (ΔABR) and AJBR (ΔAJBR) was 2.71 (SD = 9.40; ranging from − 20 to 30) and 1.89 (SD = 8.86; ranging from − 22 to 38).

### Correlational analyses

Table 4 describes the correlations between mean scores and bleeding rates. As expected, a negative relation was apparent between ABR, AJBR, ΔABR, ΔAJBR and scores. The parent proxy report CHO-KLAT was most strongly related to the ABR (*r* = − 0.609, *p* = 0.000), AJBR (*r* = − 0.353, *p* = 0.030) and ΔABR (*r* = − 0.573, *p* = 0.000). Additionally, a significantly negative correlation was found between SEC parent proxy report scores and ABR, ΔABR, ΔAJBR (*r* = − 0.569, *p* = 0.000 and *r* = − 0.538, *p* = 0.000 and *r* = − 0.381 *p* = 0.018, respectively). There was no correlation between bleeding rates and child self-report scores of CHO-KLAT. Pearson bivariate correlations revealed positive significant association between SEC and patient HRQoL regardless of baseline or follow-up. The follow-up results showed that child self-reports had higher correlations with parent proxy reports in CHO-KLAT (*r* = 0.772) and SEC (*r* = 0.690). The correlation analysis indicated that SEC showed intimate correlations with the CHO-KLAT, suggesting the SEC module was necessary for Chinese patients.

**Table 4:** Correlations between Scores at follow-up and ABR and AJBR

	CHOKLAT Child self-report		CHOKLAT Parent proxy report		SEC Child self-report		SEC Parent proxy report	
	*r*	*P* ^*1*^	*r*	*P* ^*1*^	*r*	*P* ^*1*^	*r*	*P* ^*1*^
ABR ^1^	− 0.188	0.454	**-0.609** ^**2**^	**0.000** ^**2**^	− 0.241	0.336	**-0.569** ^**2**^	**0.000** ^**2**^
AJBR^1^	− 0.112	0.659	**-0.353** ^**3**^	**0.030** ^**3**^	− 0.014	0.955^3^	− 0.251	0.128
ΔABR^1^	− 0.314	0.205	**-0.573** ^**2**^	**0.000** ^**2**^	**-0.537** ^**3**^	**0.021** ^**3**^	**-0.538** ^**2**^	**0.000** ^**2**^
ΔAJBR^1^	− 0.129	0.610	−0.273	0.097	−0.287	0.249	**-0.381** ^**3**^	**0.018** ^**3**^

*ABR* annualized bleeding rate, *AJBR* annualized joint bleeding rate

^1^Correlated by the Spearman correlation

^2^Correlation is significant at the 0.01 level (2-tailed)

^3^Correlation is significant at the 0.05 level (2-tailed)

Value in bold indicate significant *P* value

### Prognosis analyses

**Fig. 1:**
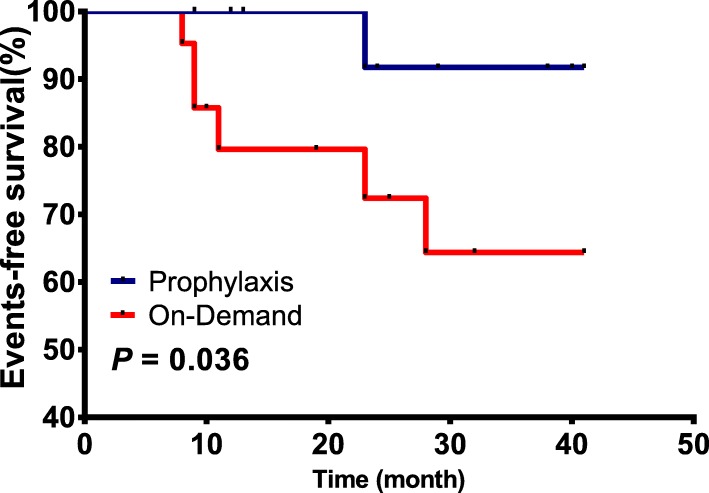
Kaplan-Meier Survival Curves by Treating Method. The treating method curves were significantly different from each other (*p* = 0.036)

The events we considered here were death, target joints developing and severe bleeding. Throughout the follow up, 5 special cases were recorded. One of them suffered from severe bleeding (urinary system) and the rest developed new target joints. The 5 cases observed were all haemophilia A, severe in 3, moderate in 1 and mild in 1. When it comes to treatment assessment, 4 cases received on-demand treatment whereas only 1 case received prophylaxis treatment. In a comprehensive multivariate Cox regression analysis including age and region of residence, the treating method remained an independent prognostic variable for haemophilia (*P* = 0.036, Fig. 1). Prophylaxis treatment is a key factor contributing to prognosis.

## Discussion

This study demonstrated that haemophilia decreased HRQoL of patients, but this effect weakened at year 4 compared with the baseline, the primary outcome. In addition, exploratory secondary outcomes also showed that HRQoL was influenced by bleeding rates, physical activity restriction, financial burden and treatment. Prophylactic treatment was a key factor contributing to event-free survivor prognosis and an optimal therapy for haemophilia patients.

To our knowledge, this was the first long-time follow-up prospective cohort study to estimate the potential factor influencing haemophilia HRQoL and to provide a direct comparison of prognosis for treatment (on-demand vs prophylaxis treatment) in haemophiliac children in China. We found that haemophilia could have adverse effects on functioning and well-being of children. The children and their parents usually had impaired HRQoL because of the limitations imposed on daily activity and social, emotional functioning. Facing unpredictable bleeding events, the families showed greater concern, which affected the whole families’ QoL as a consequence. However, when the children grew up, they could be less worried, with a full knowledge of haemophilia and better preparation for bleeding events. As life expectancy among haemophilia has increased, stable partnerships have become prominent features in the life planning of haemophilia, also improving HRQoL.

Mean value of CHO-KLAT parent proxy reports (64.69, SD = 13.71) was similar to child self-reports (65.33, SD = 15.78) without statistically significant differences. This result is comparable to the study of L. Tang et al. in China [16], but the scores were significantly lower than reported scores in developed countries such as Canada [18](75.4, SD = 12.0), Germany (71.5, SD = 13.3), France (76.7, SD = 9.7), Spain (78.4, SD = 10.8) and the Netherlands (82.8, SD = 8.4) [14], where prophylaxis is taken as the standard treatment of haemophilia with severe haemophilia. This difference indicates that economic development may have a considerable effect on the QoL of haemophilia. In this study, haemophilia patients living in urban areas had higher average HRQoL values than in rural areas. As is known, the high factor of consumption and cost presents a major barrier to haemophilia treatment in developing countries [20]. Patients living in urban areas like Nanjing city have a higher medical insurance covering 85% of the total medical cost. But in rural areas, patients are more constrained by their financial status with a lower medical insurance coverage, varying from 30 to 75% in different areas. Financial burden affects patients’ HRQoL. Meanwhile, the SEC, which added important additional information related to the social and economic context of the patient, also supported this conclusion.

QoL is a subjective assessment affected by multiple factors. As can be seen from Table 4, bleeding rates were proportionally negatively correlated with HRQoL. Furthermore, the obvious correlation in the parents’ reports showed their greater concern towards the bleeding issue. Repeated joint bleeding, depending on the severity of the disease, can cause severe joint damage, pain, and disability [21]. It might be expected that individuals disabled by dysfunctional joints and chronic pain would encounter problems in landing a job, seeking a partner, or having children. Therefore, most of the patients and their parents feel anxiety towards their uncertain future. Many daily activities are limited to avoid bleeding events, which might influence the children’s QoL as well. At the same time, patients who had received home injection got higher scores, indicating that timely solution of factor replacement could reduce their concern, improving QoL.

Our data showed that HRQoL scores were higher in children who had received prophylaxis compared to on-demand treatment. Earlier implementation of prophylaxis treatment is known to decrease bleeding rates to protect joints from damage by routine replacement of deficient clotting factor [6, 21]. Our patients in this study received medium dose prophylaxis treatment, which was used since the 1960s in Netherlands with a dose of 15–25 IU/kg every other day or three times a week [22]. Most of the patients began prophylaxis when they were younger than 3, which may protect and improve HRQoL. Table 4 reveals an inconsistency between the average scores of the parent proxy report and child self-report groups. As Rama Zilber mentioned, the basic concern of all parents was the health and future of their children [23]. Yet the life experiences and inner lives of children were different from those of the adults, resulting them having different priorities, as reflected in the scores. No significant relation was found between the type of haemophilia and HRQoL. The effect of an inhibitor was not addressed in the study since there was only 1 respondent.

This work also demonstrated that 17 patients undergoing primary prophylaxis treatment took a lower risk to develop target joints and suffer severe bleeding. Many studies have revealed early high-dose prophylaxis provides excellent long-term clinical outcomes [24]. The World Health Organization (WHO) and the World Federation of Hemophilia (WFH) suggest that initiating prophylactic treatment at an early age is the optimal form of therapy for a child with severe haemophilia [2, 5]. 13 of the 17 patients in this study had received prophylactic treatment before the age of 3. Only 1 case in the prophylactic treatment group developed a target joint during the follow-up period, and no one suffered from severe bleeding.

Some limitations of the study should be addressed. Firstly, the study was limited by a small sample size of subjects. Children need to be older than 7 to finish the self-reporting part independently, yet the median age of haemophilia children here was 5.48 years old, which severely reduces the number of qualified subjects. It is essential to validate the results in a larger size of subjects. Secondly, this study focused only on the factor replacement treatment rather than some new treatment. Chinese parents prefer taking traditional Chinese medicines concurrently. Future research should take the limitations above into account.

## Conclusions

Overall, haemophilia has an impact on the HRQoL of patients, who had a lower increase in HRQoL after 4 years. HRQoL of haemophilia is influenced by severity of disease, bleeding rates, physical activity restriction, financial burden and treatment. Prophylactic treatment is an optimal therapy for childhood haemophilia and a key factor contributing to event-free survival prognosis.

## Abbreviations

ABR: Annual bleeding rate
AJBR: Annual joint bleeding rate
CHO-KLAT: Canadian Haemophilia Outcomes–Kids’ Life Assessment Tool
HRQoL: Health-related Quality of Life
SEC: Socio-Economic Context
SPSS: Statistical Package for Social Science
